# Assay optimization for molecular detection of Zika virus

**DOI:** 10.2471/BLT.16.175950

**Published:** 2016-12-01

**Authors:** Victor M Corman, Andrea Rasche, Cecile Baronti, Souhaib Aldabbagh, Daniel Cadar, Chantal BEM Reusken, Suzan D Pas, Abraham Goorhuis, Janke Schinkel, Richard Molenkamp, Beate M Kümmerer, Tobias Bleicker, Sebastian Brünink, Monika Eschbach-Bludau, Anna M Eis-Hübinger, Marion P Koopmans, Jonas Schmidt-Chanasit, Martin P Grobusch, Xavier de Lamballerie, Christian Drosten, Jan Felix Drexler

**Affiliations:** a Institute of Virology, University of Bonn Medical Centre, Sigmund Freud-Str. 25, 53127 Bonn, Germany.; b UMR EPV Emergence des Pathologies Virales, Aix Marseille Université, Marseille, France.; c Bernhard Nocht Institute for Tropical Medicine, WHO Collaborating Centre for Arbovirus and Hemorrhagic Fever Reference and Research, Hamburg, Germany.; d Erasmus MC, Department of Viroscience, Rotterdam, Netherlands.; e Department of Infectious Diseases, University of Amsterdam, Amsterdam, Netherlands.; f Clinical Virology Laboratory, University of Amsterdam, Amsterdam, Netherlands.

## Abstract

**Objective:**

To examine the diagnostic performance of real-time reverse transcription (RT)-polymerase chain reaction (PCR) assays for Zika virus detection.

**Methods:**

We compared seven published real-time RT–PCR assays and two new assays that we have developed. To determine the analytical sensitivity of each assay, we constructed a synthetic universal control ribonucleic acid (uncRNA) containing all of the assays’ target regions on one RNA strand and spiked human blood or urine with known quantities of African or Asian Zika virus strains. Viral loads in 33 samples from Zika virus-infected patients were determined by using one of the new assays.

**Findings:**

Oligonucleotides of the published real-time RT–PCR assays, showed up to 10 potential mismatches with the Asian lineage causing the current outbreak, compared with 0 to 4 mismatches for the new assays. The 95% lower detection limit of the seven most sensitive assays ranged from 2.1 to 12.1 uncRNA copies/reaction. Two assays had lower sensitivities of 17.0 and 1373.3 uncRNA copies/reaction and showed a similar sensitivity when using spiked samples. The mean viral loads in samples from Zika virus-infected patients were 5 × 10^4^ RNA copies/mL of blood and 2 × 10^4^ RNA copies/mL of urine.

**Conclusion:**

We provide reagents and updated protocols for Zika virus detection suitable for the current outbreak strains. Some published assays might be unsuitable for Zika virus detection, due to the limited sensitivity and potential incompatibility with some strains. Viral concentrations in the clinical samples were close to the technical detection limit, suggesting that the use of insensitive assays will cause false-negative results.

## Résumé

**Objectif:**

Étudier la performance diagnostique des tests basés sur l'amplification en chaîne par polymérase (PCR) en temps réel après transcription inverse (RT) pour détecter le virus Zika.

**Méthodes:**

Nous avons comparé sept tests publiés utilisant la RT-PCR en temps réel et deux nouveaux tests développés par nos soins. Afin de déterminer la sensibilité analytique de chaque test, nous avons conçu un acide ribonucléique synthétique de contrôle universel (ARNcun) contenant toutes les régions ciblées par les tests sur une hélice ARN et enrichi de l’urine ou du sang humain de quantités connues de souches africaines ou asiatiques du virus Zika. Les charges virales dans 33 échantillons provenant de patients infectés par le virus Zika ont été déterminées à l'aide de l'un des nouveaux tests.

**Résultats:**

Les oligonucléotides des tests publiés utilisant la RT-PCR en temps réel ont présenté jusqu'à 10 mauvais appariements potentiels avec la lignée asiatique provoquant l'épidémie actuelle, alors qu'on a constaté de 0 à 4 mauvais appariements dans le cas des nouveaux tests. La limite de détection inférieure à 95% des sept tests les plus sensibles variait de 2,1 à 12,1 copies d'ARNcun/réaction. Deux tests présentaient des sensibilités plus basses de 17,0 et 1373,3 copies d'ARNcun/réaction et montraient une sensibilité similaire lorsque des échantillons enrichis étaient utilisés. Les charges virales moyennes dans les échantillons provenant de patients infectés par le virus Zika étaient de 5 × 10^4^ copies d'ARN/mL de sang et de 2 × 10^4^ copies d'ARN/mL d'urine.

**Conclusion:**

Nous proposons pour détecter le virus Zika des réactifs et des protocoles actualisés, adaptés aux souches responsables de la flambée actuelle. Tous les tests publiés ne permettent pas de détecter le virus Zika en raison d'une sensibilité limitée et d'une incompatibilité potentielle avec certaines souches. Les concentrations virales dans les échantillons cliniques étaient proches de la limite de détection technique, ce qui laisse penser que l'utilisation de tests insensibles donnera des résultats faussement négatifs.

## Resumen

**Objetivo:**

Examinar el rendimiento del diagnóstico de las pruebas de reacción en cadena de la polimerasa de transcriptasa inversa (RT-PCR) en tiempo real para la detección del virus de Zika.

**Métodos:**

Se compararon siete pruebas de RT-PCR en tiempo real publicadas y dos pruebas nuevas que se han desarrollado. Con el fin de determinar la sensibilidad analítica de cada prueba, se construyó un ácido ribonucleico sintético de control universal (uncRNA) que contenía todas las regiones objetivo de las pruebas en una hebra de RNA y se añadió a sangre u orina humana con cantidades conocidas de cepas de virus de Zika de Asia o África. Se determinaron las cargas víricas en 33 muestras procedentes de pacientes infectados por el virus de Zika a través de una de las pruebas nuevas.

**Resultados:**

Los oligonucleótidos de las pruebas de RT-PCR en tiempo real publicadas mostraron hasta 10 posibles discordancias con el linaje de Asia causante de los brotes actuales, en comparación con 0 de 4 discordancias en el caso de las pruebas nuevas. El límite de detección inferior del 95% de las siete pruebas más sensibles abarcó de 2,1 a 12,1 copias/reacción de uncRNA. Dos pruebas mostraron sensibilidades inferiores de 17,0 y 1373,3 copias/reacción de uncRNA y presentaron una sensibilidad similar al usar muestras infectadas. Las cargas víricas medias en las muestras procedentes de pacientes infectados por el virus de Zika fueron de 5 × 10^4^ copias de RNA/mL de sangre y de 2 × 10^4^ copias de RNA/ml de orina.

**Conclusión:**

Se proporcionan reactivos y protocolos actualizados para la detección adecuada del virus de Zika en el caso de las cepas de brotes actuales. Algunas pruebas publicadas pueden no ser adecuadas para la detección del virus de Zika, debido a la limitada sensibilidad y a la posible incompatibilidad con algunas cepas. Las concentraciones víricas en las muestras clínicas se acercaron al límite de detección técnico, lo que sugería que el uso de pruebas intensivas causaría resultados falsos negativos.

## ملخص

**الغرض:**

دراسة الأداء التشخيصي لاختبارات تفاعل البوليميراز المتسلسل اللحظي (PCR) مع إنزيم النسخ العكسي (RT) لاكتشاف الإصابة بفيروس زيكا.

**الطريقة:**

قمنا بإجراء مقارنة بين سبعة اختبارات منشورة لتفاعل البوليميراز المتسلسل اللحظي مع إنزيم النسخ العكسي (RT‑PCR) واختبارين جديدين تم إعدادهما من جانبنا. ولتحديد الحساسية التحليلية لكل اختبار، قمنا بتكوين الحمض النووي الريبوزي الاصطناعي للمكافحة العامة (uncRNA) والذي يحتوي على جميع المناطق المستهدفة في الاختبار في شريط RNA واحد ويحتوي على مؤشرات للدم أو البول البشري الذي يحتوي على كميات معروفة من سلالات فيروس زيكا في منطقة أفريقيا أو آسيا. وتم تحديد الحمولات الفيروسية في 33 عينة صادرة من مرضى مصابين بفيروس زيكا باستخدام أحد الاختبارات الجديدة.

**النتائج:**

أظهرت الأوليغونيكليوتيدات الخاصة باختبارات تفاعل البوليميراز المتسلسل اللحظي مع إنزيم النسخ العكسي ما يصل إلى 10 حالات محتملة من عدم التطابق مع السلالة الآسيوية المسببة للعدوى الحالية، بالمقارنة مع حالات عدم التطابق بالنسبة للاختبارات الجديدة بمعدل 0 إلى 4. تراوح حد الاكتشاف المنخفض بنسبة 95‏% في الاختبارات السبعة الأكثر حساسية من 2.1 إلى 12.1 من نسخ سلالات uncRNA لتفاعل كل حالة. وُجد لدى اثنين من الاختبارات درجات منخفضة من الحساسية بمقدار 17.0 و1373.3 لنسخ سلالات uncRNA لتفاعل كل حالة وأظهرت وجود درجة حساسية مماثلة عند استخدام عينات المؤشرات. بلغ متوسط الحمولات الفيروسية في العينات الصادرة عن المرضى المصابين بفيروس زيكا 5 × 10 ^4 ^ نسخ من RNA لكل ملليلتر من الدم و 2 × 10 ^4 ^ نسخ من RNA لكل ملليلتر من البول.

**الاستنتاج:**

نحن نوفر الكاشفات وأحدث البروتوكولات لاكتشاف فيروس زيكا والتي تناسب سلالات العدوى الحالية. وقد تكون بعض الاختبارات المنشورة غير مناسبة لاكتشاف فيروس زيكا نظرًا لوجود درجة محدودة من الحساسية واحتمالية عدم توافقها مع بعض السلالات. كانت نسب تركيز الفيروسات في العينات التحليلية قريبة من حد الاكتشاف التقني، مما يشير إلى أن استخدام الاختبارات التي تفتقد إلى الحساسية سيؤدي إلى إصدار نتائج خاطئة وسلبية.

## 摘要

**目的:**

旨在检查针对寨卡病毒检测的实时逆转录 (RT)-聚合酶链反应 (PCR) 试验的诊断性能。

**方法:**

我们比较了 7 种公布的实时 RT-PCR 试验和我们开发的两种新试验。 为了确定各种试验的分析灵敏度，我们构建了在一个 RNA 链上包含所有试验目标区域的合成通用对照核糖核酸 (uncRNA) 以及带有已知数量的非洲或亚洲寨卡病毒株的加标人体血液或尿液。 通过使用一种新的试验测定 33 个寨卡病毒感染患者样本的病毒载量。

**结果:**

已公布的实时 RT-PCR 试验的寡核苷酸，显示出与亚洲血统潜在的失配率高达 10，相比之下，新试验的失配率为 0 至 4。 七种最敏感试验的 95% 检测下限范围为 2.1 至 12.1 uncRNA 拷贝/反应。 两个试验中 17.0 和 1373.3 uncRNA 复制/反应的灵敏度较低，使用加标样本时表现出类似的灵敏度。 寨卡病毒感染患者的样本中，血液的平均病毒载量为 5 × 10^4^ RNA 拷贝/毫升，尿液的平均病毒载量为 2 × 10^4^ 拷贝/毫升。

**结论:**

我们为适合当前疫情的寨卡病毒检测提供试剂和更新方案。 由于有限的灵敏度以及与一些菌株潜在的不兼容性，因此一些公布的试验可能不适合寨卡病毒检测。 临床样本中的病毒含量接近于技术检测极限，这表明使用不敏感试验将会引起假阴性结果。

## Резюме

**Цель:**

Изучить диагностические возможности выявления вируса Зика с помощью полимеразной цепной реакции (ПЦР) с обратной транскриптазой (ОТ) в режиме реального времени.

**Методы:**

Авторы сравнили семь методов ОТ-ПЦР, сведения о которых были опубликованы в литературе, и два новых метода, разработанные авторами. Для выявления аналитической чувствительности каждого метода были сконструированы синтетические универсальные контрольные рибонуклеиновые кислоты (ункРНК), содержащие все целевые участки на одной из цепей РНК. Эти РНК были добавлены к образцам крови или мочи пациентов, содержащих вирус Зика африканского или азиатского происхождения в известных количествах. Одним из новых методов определялась вирусная нагрузка для 33 образцов пациентов, зараженных вирусом Зика.

**Результаты:**

Олигонуклеотиды для опубликованных методов ОТ-ПЦР показали до 10 потенциальных несовпадений при определении вируса азиатского происхождения, вызвавшего нынешнюю вспышку заболеваемости, тогда как новые методы показали от 0 до 4 несовпадений. Для семи наиболее чувствительных анализов 95%-й нижний порог определения составлял от 2,1 до 12,1 копии ункРНК на реакцию. Два метода продемонстрировали меньшую чувствительность (от 17,0 до 1373,3 копии ункРНК на анализ) и сходную чувствительность при использовании образцов с добавлением ункРНК. Средняя величина вирусной нагрузки в образцах пациентов, инфицированных вирусом Зика, составила 5 × 10^4^ копий РНК/мл для крови и 2 × 10^4 ^копийРНК/мл для мочи.

**Вывод:**

Авторы предлагают реактивы и обновленные протоколы для выявления вируса Зика, вызвавшего нынешнюю вспышку заболевания. Некоторые из методов, по которым имеются опубликованные литературные данные, могут быть неподходящими для текущей ситуации из-за ограниченной чувствительности и потенциальной несовместимости с некоторыми штаммами вируса. Концентрация вируса в клинических образах была близка к техническому пределу определения, что позволяет предположить, что использование нечувствительных методов может приводить к получению ложноотрицательных результатов.

## Introduction

The Zika virus is a mosquito-borne flavivirus with an approximately 11 kilobase ribonucleic acid (RNA) genome.^1^ The virus usually causes a mild infection in adults, symptoms include fever, arthralgia and rash.^2^^,^^3^ However, severe complications can occur, such as Guillain-Barré syndrome,^4^ meningoencephalitis,^5^ hearing loss and uveitis.^6^^,^^7^ In the current Zika virus outbreak, intrauterine infections have been associated with fetal malformations.^8^^–^^11^

Reliable detection of the Zika virus in infected people is key to understanding the epidemiology, the pathogenesis and alternative transmission routes of the virus, such as sexual intercourse and blood transfusions.^12^ However, in areas where the Zika virus is co-circulating with dengue and chikungunya viruses, physicians cannot reliably diagnose the Zika virus infection by clinical presentation, because the viruses cause similar symptoms. Using serology for Zika virus diagnostics can be challenging because of the cross-reactivity of antibodies elicited by other endemic flaviviruses – such as dengue, yellow fever, St Louis encephalitis and West Nile viruses.^3^^,^^13^^,^^14^ Molecular detection of viral nucleic acid using real-time reverse transcription (RT)-polymerase chain reaction (PCR) assay is a highly reliable diagnostic method during acute infection. Currently, there are six widely used real-time RT–PCR assays for Zika virus detection.^7^^,^^13^^,^^15^^,^^16^ The Pan American Health Organization (PAHO) has recommended an additional real-time RT–PCR assay.^12^

**Fig. 1:**
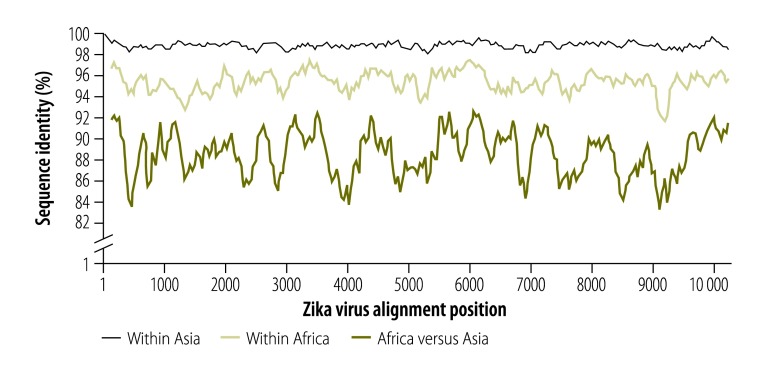
Zika virus genomic identity plot

Notes: Identity plot of all Zika virus polyprotein sequences characterized to at least 80% available at GenBank® by 13 April 2016. Similarity plots were done using SSE V1.2.^18^ A sliding window of 200 and a step size of 40 nucleotides were used.

**Fig. 2:**
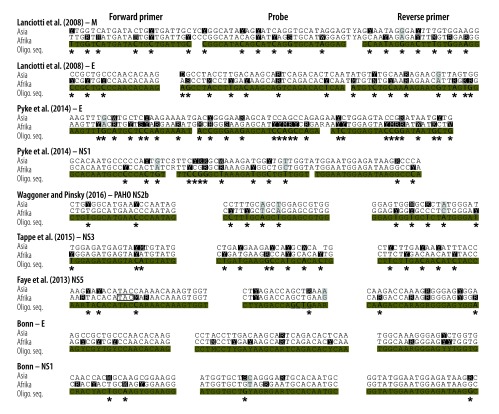
**Alignment of real-time reverse transcription polymerase chain reaction assays and Zika virus lineages**

High real-time RT–PCR sensitivity is important to avoid false-negative results. Nucleotide mutations in the binding sites of primers and probes can affect the sensitivity.^17^ So far, the genetic variability of the Zika virus Asian lineage causing the current American outbreak is limited to about 2% nucleotide differences across the viral genome (Fig. 1). However, mutations do not occur evenly across viral genomes. Up to 10 nucleotide mismatches between the oligonucleotide sequences of the published assays and the Asian lineage consensus sequence already exist and in individual primers or probes, there are up to five mismatches (Fig. 2). Note that these are worst-case scenarios based on the genetic variability permitted within the Asian Zika virus lineage, with no single known Zika virus strain accumulating all of these mismatches. However, the increasing number of divergent Zika virus outbreak strains highlights the genetic variability as a potential limiting factor of the sensitivity of Zika virus real-time RT–PCR-based diagnostics.

E: envelope; M: membrane; NS: nonstructural protein;

Oligo: oligonucleotide; PAHO: Pan American Health Organization; PCR: polymerase chain reaction; RNA; ribonucleic acid; seq: sequence.

Notes: For each of the African and Asian lineage, 100% consensus sequences were generated and mapped to respective PCR primers and probes. Consensus calculations included all 259 complete and partial Zika virus sequences available at GenBank® by 7 April 2016. Variable sites within the oligonucleotide binding sites of the two Zika virus lineages are highlighted in black, variable sites between the two lineages in grey. Potential mismatches in oligonucleotides are indicated by asterisks. Y = C/T, R = A/G, M = A/C, B = C/G/T, S = G/C, W = A/T, H = A/C/T, D = A/G/T, *n* = A/C/T/G, K = G/T, V = A/C/G. Locked nucleic acids in the probe of the Faye et al.^15^ NS5-based assay are underlined and a three nucleotide deletion in some African virus strains is boxed.

Here, we compare the sensitivity of published real-time RT–PCR assays and two new assays, which we designed to have less nucleotide mismatches with the current outbreak strains. We also present data on viral load profiles in blood and urine from infected patients, using one of the new assays. 

## Methods

### Assays

We compared nine different assays. We included all Zika virus real-time RT–PCR assays published until 1 April 2016. These assays target the membrane (M), envelope (E), nonstructural protein (NS) 1, NS2b, NS3 and NS5 genomic domains.^7^^,^^12^^,^^13^^,^^15^^,^^16^ We designed two new assays covering the currently known Zika virus genetic variability in the E and NS1 genomic domains (Table 1). These novel assays showed up to four potential mismatches per assay (Fig. 2) and were designed to avoid mismatches in the most critical 3′-terminal regions of oligonucleotides that affect primer binding the most.^17^^,^^19^ The new NS1 assay was additionally designed to allow cross-detection of the Spondweni virus – the closest relative of the Zika virus – because regions conserved between related virus taxa are expected to have less variation than other genomic regions.

**Table 1:** Oligonucleotides used in Zika virus real-time reverse transcription polymerase chain reaction assays and potential nucleotide mismatches with Zika virus strains

Assay, reference	Target genomic domain (bases)^a^	No. potential nucleotide mismatches^a^		Forward primer sequence (5‘ to 3‘)	Probe sequence (5‘ to 3‘)^b^	Reverse primer sequence (5‘ to 3‘)	Control (covered genomic region, bases)^a^
		All Zika virus	Asian lineage				
Lanciotti M^13^	M/E (939–1015)	19	7	TTGGTCATGATACTGCTGATTGC	CGGCATACAGCATCAGGTGCATAGGAG	CCTTCCACAAAGTCCCTATTGC	uncRNA; IVT I (811–1500)
Lanciotti E^13^	E (1190–1266)	18	4	CCGCTGCCCAACACAAG	AGCCTACCTTGACAAGCAGTCAGACACTCAA	CCACTAACGTTCTTTTGCAGACAT	uncRNA; IVT I (811–1500)
Bonn E (this study)	E (1188–1316)	0	0	AGYCGYTGYCCAACACAAG	CCTMCCTYGAYAAGCARTCAGACACYCAA	CACCARRCTCCCYTTGCCA	uncRNA; IVT I (811–1500)
Pyke E^16^	E (1326–1397)	28	7	AAGTTTGCATGCTCCAAGAAAAT	ACCGGGAAGAGCATCCAGCCAGA	CAGCATTATCCGGTACTCCAGAT	uncRNA; IVT I (811–1500)
Pyke NS1^16^	NS1 (3433–3498)	13	7	GCACAATGCCCCCACTGT	TTCCGGGCTAAAGATGGCTGTTGGT	TGGGCCTTATCTCCATTCCA	uncRNA; IVT II (3 145–3739)
Bonn NS1 (this study)	NS1 (3385–3495)	4	3	CRACYACTGCAAGYGGAAGG	ATGGTGCTGYAGRGARTGCACAATGC	GCCTTATCTCCATTCCATACC	uncRNA; IVT II (3145–3739)
PAHO NS2b^12^	NS2b/NS3 (4538–4628)	11	4	CTGTGGCATGAACCCAATAG	CCACGCTCCAGCTGCAAAGG	ATCCCATAGAGCACCACTCC	uncRNA; IVT III (4246–4882)
Tappe NS3^7^	NS3 (6012–6106)	15	10	TGGAGATGAGTACATGTATG	CTGATGAAGGCCATGCACACTG	GGTAGATGTTGTCAAGAAG	uncRNA; IVT IV ( 770–6370)
Faye NS5^15^	NS5 (9376–9477)	6	3	AARTACACATACCARAACAAAGTGGT	CTYAGACCA**GCTG**AAR^c^	TCCRCTCCCYCTYTGGTCTTG	uncRNA; IVT V (9100–9696)
Marker assay 1 (this study)	M/NS1	N/A	N/A	GCATCCAGCCAGAGAATCTG	TGCTGTCAGTTCACTCAAGGTTAGAGA	CAATAACGGCTGGATCACACTC	uncRNA (N/A)
Marker assay 2 (this study)	NS3/NS4b-NS5	N/A	N/A	CTTGACAATATTTACCTCCAAGATG	CATAGCCTCGCTCTCTACACATGAGA	GTTGCTTTTCGCTCCAGAGAC	uncRNA (N/A)

E: envelope; IVT: in vitro transcribed ribonucleic acid; M: membrane; N/A: not applicable; NS: nonstructural protein; PAHO: Pan American Health Organization; uncRNA: universal control ribonucleic acid.

^a^ Nucleotide position according to GenBank® accession number: KU321639.

^b^ All probes are labelled with fluorescein amidite (FAM) at the 5′-end and a Black Hole Quencher® at the 3′-end.

^c^ Locked nucleic acid bases are underlined.

### Controls

**Fig. 3:**
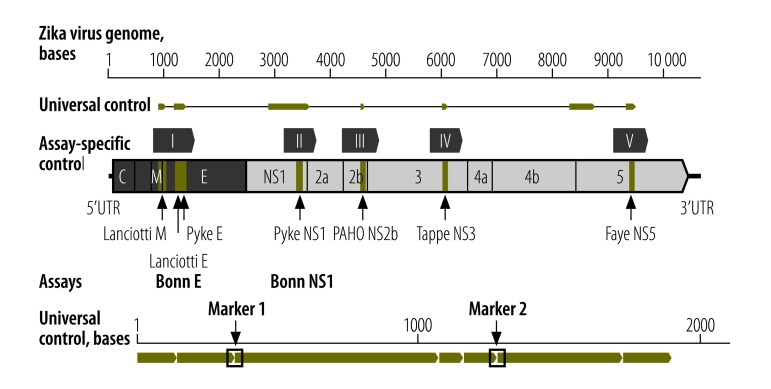
**Genomic locations of oligonucleotides and controls used in real-time reverse transcription polymerase chain reaction assays for Zika virus detection**

All controls are based on a current Zika virus outbreak strain (GenBank® accession number KU321639). As positive controls, we generated five assay-specific quantified in vitro transcripts (IVT) for the respective genomic target regions. Data on analytical sensitivity, including the standardized measure lower limit of detection, are not available for most of the published assays. To enable stoichiometrically exact analyses of the lower limit of detection for all of the assays, we joined all target domains into a quantative universal control ribonucleic acid (uncRNA) containing all of the assays’ target regions on one RNA strand (Fig. 3; Table 1). All controls can be acquired via the European Virus Archive at the following links: Zika virus IVT I available at: http://www.european-virus-archive.com/Portal/produit.php?ref=1598&id_rubrique=9; Zika virus IVT II available at: http://www.european-virus-archive.com/Portal/produit.php?ref=1599&id_rubrique=9; Zika virus IVT III available at: http://www.european-virus-archive.com/Portal/produit.php?ref=1600&id_rubrique=9; Zika virus IVT IV available at: http://www.european-virus-archive.com/Portal/produit.php?ref=1601&id_rubrique=9; Zika virus IVT V available at: http://www.european-virus-archive.com/Portal/produit.php?ref=1602&id_rubrique=9; and uncRNA 1.0 available at: http://www.european-virus-archive.com/Portal/produit.php?ref=1603&id_rubrique=9.

E: envelope; M: membrane; NS: nonstructural protein; UTR: untranslated region.

Notes: Zika virus genomic representation (GenBank® accession no. KU321639), with real-time RT–PCR assays identified below by respective first authors or location and corresponding control in vitro transcripts (IVT) and parts of the universal control ribonucleic acid (uncRNA) identified above. Genomic regions not containing published assays so far, but potentially useful for future assay design due to genomic conservation within the Asian Zika virus lineage were also included in the uncRNA. Lower panel, representation of uncRNA and the marker assays, which can detect potential cases of laboratory contamination. The lower limit of detections of the two marker assay variants (both can be used interchangeably for confirmatory analyses) were comparable to Zika virus-specific assays with 4.3 (95% confidence interval, CI: 2.9–10.9) and 3.3 (95% CI: 2.4–6.5) copies/reaction, respectively. Already published assays are Lanciotti M,^13^ Lanciotti E,^13^ Pyke E,^16^ Pyke NS1,^16^ PAHO NS2b,^12^ Tappe NS3^7^ and Faye NS5.^15^

The uncRNA was generated as described previously.^20^ In brief, the uncRNA was custom designed as a gBlocks® fragment with a T7 promotor sequence (Integrated DNA Technologies, Leuven, Belgium) and in vitro transcribed.^20^

A disadvantage of using a test control with a high concentration of viral RNA (e.g. from cell culture) is the potential for laboratory contamination, potentially causing false-positive test results. In contrast to natural viral RNA, potential cases of laboratory contamination with the uncRNA can be proven by two real-time RT–PCR marker assays we designed specifically to detect the uncRNA (Table 1). These two marker assays contain detection probes that target the overlap of two joined genomic target domains, which do not naturally occur in the Zika virus genome (Fig. 3).

### Quantification and characterization

We purified viral RNA using the Qiagen Viral RNA Mini Kit (Qian, Hilden, Germany) or the MagNA Pure 96 Viral NA Small Volume Kit (Roche, Basel, Switzerland) according to the manufacturer's instructions. Dengue virus RNA quantification and flavivirus typing were done as described previously.^21^^,^^22^

For all of the experiments, except when assessing threshold cycle variation using different reaction conditions and thermocyclers, we quantified Zika virus RNA using the LightCycler® 480 Instrument II (Roche, Basel, Switzerland). Generally, 25 μl reactions were set up with 5 μl of RNA; 12.5 μl of 2 × reaction buffer from the Superscript® III one step RT–PCR system with Platinum® Taq polymerase (Thermo Fisher Scientific, Darmstadt, Germany); 0.4 μl of a 50 mM magnesium sulfate solution (Thermo Fisher Scientific); 1 μg of nonacetylated bovine serum albumin (Roche), 600 nM of each primer and 280 nM of each probe and 1 μl of SuperScript® III RT/Platinum® Taq mix. Amplification involved 50 °C for 15 minutes, followed by 95 °C for 3 minutes and 45 cycles of 95 °C for 15 seconds, 56 °C for 20 seconds and 72 °C for 15 seconds. For comparison of C_T_ values using different PCR cyclers and chemistry, we used the Bonn E- and NS1-based assays using either the Superscript III One-Step RT–PCR kit (Thermo Fischer) or the Qiagen® One-Step RT–PCR kit (Qiagen) on a Roche LightCycler® 480 and LightCycler 2.0, a Qiagen Rotor Gene HQ and an Applied Biosystems 7500 thermocycler. Reference conditions refer to the usage of Life Technologies SuperScript® III One-Step enzyme mix and a Roche LightCycler® 480 thermocycler as described above.

Probit regression analyses, to determine the lower limit of detection for all real-time RT–PCR assays, were done using SPSS V22 (IBM, Ehningen, Germany) and eight parallel test replicates.

### Clinical specimens

We obtained clinical specimens from travellers for which routine medical investigation of either Zika or dengue virus had been requested due to compatible clinical symptoms or a travel history to affected countries. The travellers had acquired their infections in Brazil, the Dominican Republic or Suriname during 2015 and 2016.

We spiked a Zika virus-negative human plasma and urine sample with defined quantities of the African Zika virus strain MR766 and an Asian lineage outbreak strain H/PF/2013. Spiked samples were serially diluted and two replicates of each dilution were individually purified using the MagNA Pure 96 Viral NA Small Volume Kit with an input volume of 200 μL and an extraction volume of 100 μL.

## Results

All assay-specific IVT and the uncRNA allowed comparable quantification of Zika virus RNA with a mean twofold deviation of results (maximum deviation: sixfold), suggesting the ability to use these controls to generate comparable results even when different real-time RT–PCR assays are used in different laboratories. The two marker assays showed no detection of Zika virus RNA even upon using RNA from high-titred cell culture isolates (10^6^–10^9^ RNA copies/mL) as a real-time RT–PCR template.

To ensure specific detection of the Zika virus, we evaluated all assays using 37 high-titred cell culture isolates of different flaviviruses or chikungunya virus (the list of tested isolates is available from the corresponding author). None of the published assays detected the co-circulating alphavirus chikungunya virus or any other flavivirus. As expected, an Asian Zika virus lineage-specific E-based assay^16^ did not detect the African lineage despite very high concentrations of viral RNA in the sample. As intended, the new NS1-based assay cross-detected Spondweni virus, as well as Kokobera and Jugra virus. These viruses are predominantly animal-associated^23^ and do not circulate in Latin America and hence do not affect people in the current Zika virus outbreak.^14^

**Fig. 4:**
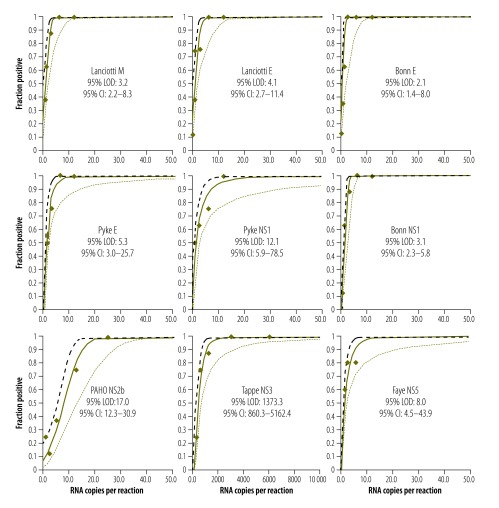
**Analytical sensitivity of Zika virus real-time reverse transcription polymerase chain reaction assays**

CI: confidence interval; LOD: lower limit of detection.

Notes: Probability of detection (*y*-axis) is plotted against universal control ribonucleic acid (uncRNA) concentration per reaction in 8 parallel test samples (*x*-axis). The diamonds represent the observed fraction of positive results in parallel experiments. Solid line, predicted proportion of positive results at a given uncRNA input concentration; dashed lines, 95% confidence interval for the prediction. Already published assays are Lanciotti M,^13^ Lanciotti E,^13^ Pyke E,^16^ Pyke NS1,^16^ PAHO NS2b,^12^ Tappe NS3^7^ and Faye NS5.^15^

When we determined the lower limit of detection using the uncRNA, all but two assays showed comparably high analytical sensitivities of around 5–10 Zika virus RNA copies/reaction (Fig. 4). An NS2b-based assay recommended by PAHO showed a lower limit of detection of 17 copies/reaction,^12^ whereas an NS3-based assay showed a lower limit of detection of 1373 copies/reaction.^7^ To exemplify the impact of technical sensitivity on clinical sensitivity, we extrapolated the lower limits of detection to clinical viral loads. As shown in Table 2, an assay with a lower limit of detection of 5–10 copies/reaction has a clinical detection limit of about 1000 copies/mL, whereas an assay with a lower limit of detection of 1000 copies/reaction has a clinical detection limit of about 10000 copies/mL. To improve clinical sensitivity, RNA purification methods could be optimized by using larger input volumes of the clinical samples (Table 2).

**Table 2:** Analytical sensitivity of Zika virus real-time reverse transcription polymerase chain reaction assays

Assay, reference	Technical 95% lower limit of detection, copies per reaction (95% CI)	Extrapolation of LOD to clinical viral loads, copies/mL	
		Assuming a 1:1 concentration during extraction^a^	Assuming a 2:1 concentration during extraction^a,b^
Lanciotti M^13^	3.2 (2.2–8.3)	640	320
Lanciotti E^13^	4.1 (2.7–11.4)	820	410
Bonn E (this study)	2.1 (1.4–8.0)	420	210
Pyke E^16^	5.3 (3.0–25.7)	1 100	530
Pyke NS1^16^	12.1 (5.9–78.5)	2 400	1 210
Bonn NS1 (this study)	3.1 (2.3–5.8)	620	310
PAHO NS2b^12^	17.0 (12.3–30.9)	3 400	1 700
Tappe NS3^7^	1 377.3 (860.3–5 162.4)	280 000	138 000
Faye NS5^15^	8.0 (4.5–43.9)	1 600	800

CI: confidence interval; E: envelope; LOD: lower limit of detection; M: membrane; RNA; ribonucleic acid; NS: nonstructural protein.

^a^ Using 5 μL of eluted ribonucleic acid per polymerase chain reaction.

^b^ Corresponding to twice higher input (e.g. 140 μl) than elution volumes (e.g. 70 μl), such as those in the commonly used Qiagen Viral RNA Mini Kit (Qiagen, Hilden, Germany), assuming a 100% extraction efficacy.

**Fig. 5:**
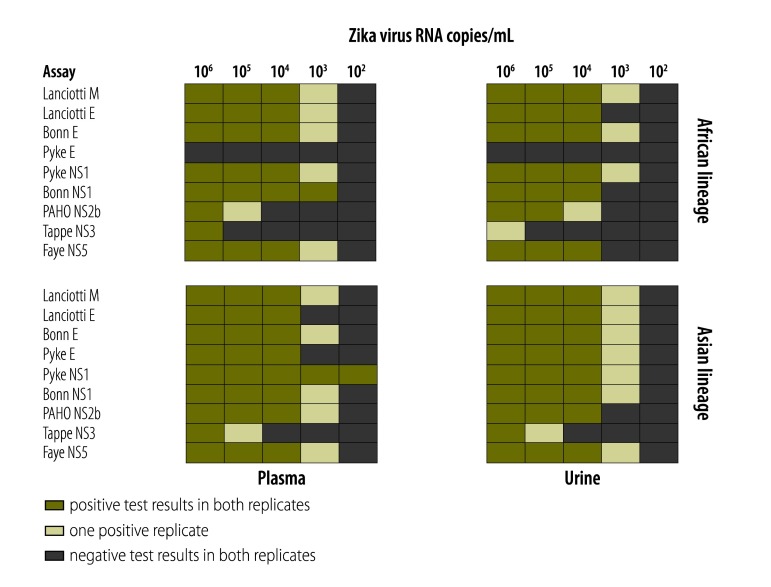
Validation of Zika virus real-time reverse transcription polymerase chain reaction assays

We examined the assays’ sensitivity in different clinical specimens by incorporating the step of RNA purification and using Zika virus-negative human blood and urine samples spiked with 10^2^–10^6^ copies/mL of either Zika virus representing the Asian lineage (strain H/PF/2013) or the African lineage (strain MR766). The results were consistent with lower limit of detections for the uncRNA. The assays with high analytical sensitivities could detect 10^3^ Zika virus RNA copies/mL of blood or urine in at least one replicate experiment, whereas the assay with the lowest analytical sensitivity yielded negative results even at high Zika virus concentrations of 10^5^ and 10^6^ copies/mL (Fig. 5).

E: envelope; M: membrane; NS: nonstructural protein; PAHO: Pan American Health Organization; RNA: ribonucleic acid.

Notes: Already published assays are Lanciotti M,^13^ Lanciotti E,^13^ Pyke E,^16^ Pyke NS1,^16^ PAHO NS2b,^12^ Tappe NS3^7^ and Faye NS5.^15^

**Fig. 6:**
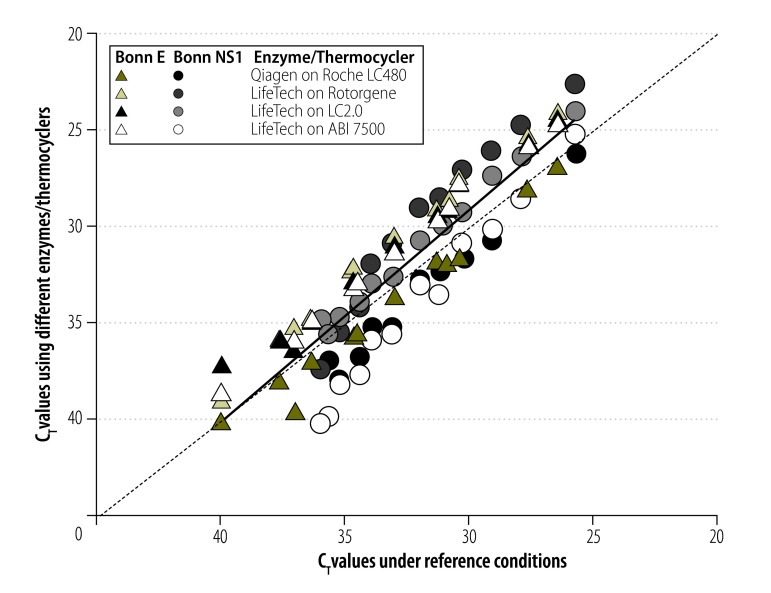
Threshold cycle variation when using different reaction conditions and thermocyclers

We explored the variability of the threshold cycle (C_T_) values – which could vary due to PCR instruments and reagents used – by testing our two new assays under diverse reaction conditions including reagents by different suppliers and different real-time RT–PCR instruments. Even a variation of only two variables could yield up to 4.3 cycles in difference in the C_T_ values for the same virus target concentration, which corresponds to about 20-fold deviations in viral load results (Fig. 6). Therefore, using C_T_ values for comparison of viral loads between studies might be misleading.^24^^,^^25^

C_T_: threshold cycle; E: envelope; NS: nonstructural protein.

Notes: Symbols identify reaction mix and thermocycler yielding different C_T_ values (*y*-axis) compared to the standard protocol and thermocycler (*x*-axis).

**Fig. 7:**
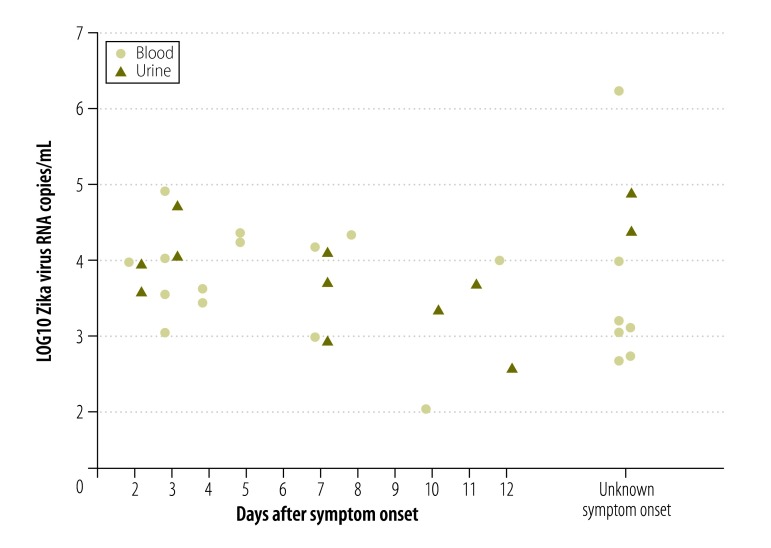
**Viral loads of Zika virus in clinical specimens**

**Fig. 8:**
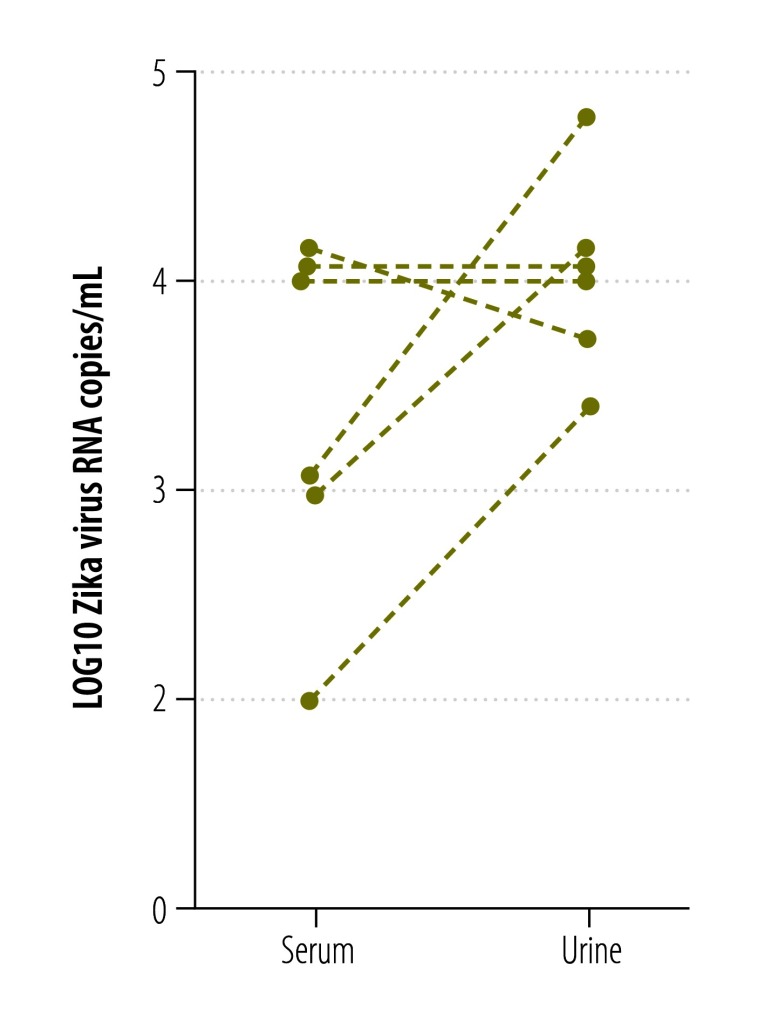
**Viral loads of Zika virus in paired urine and blood samples**

**Fig. 9:**
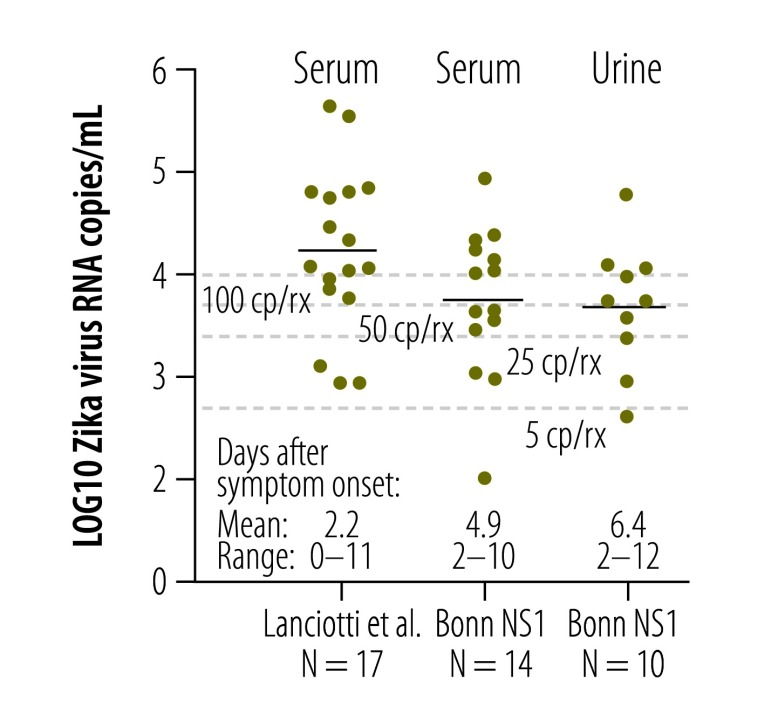
**Comparison of viral loads of Zika virus in blood and urine from different studies**

**Fig. 10:**
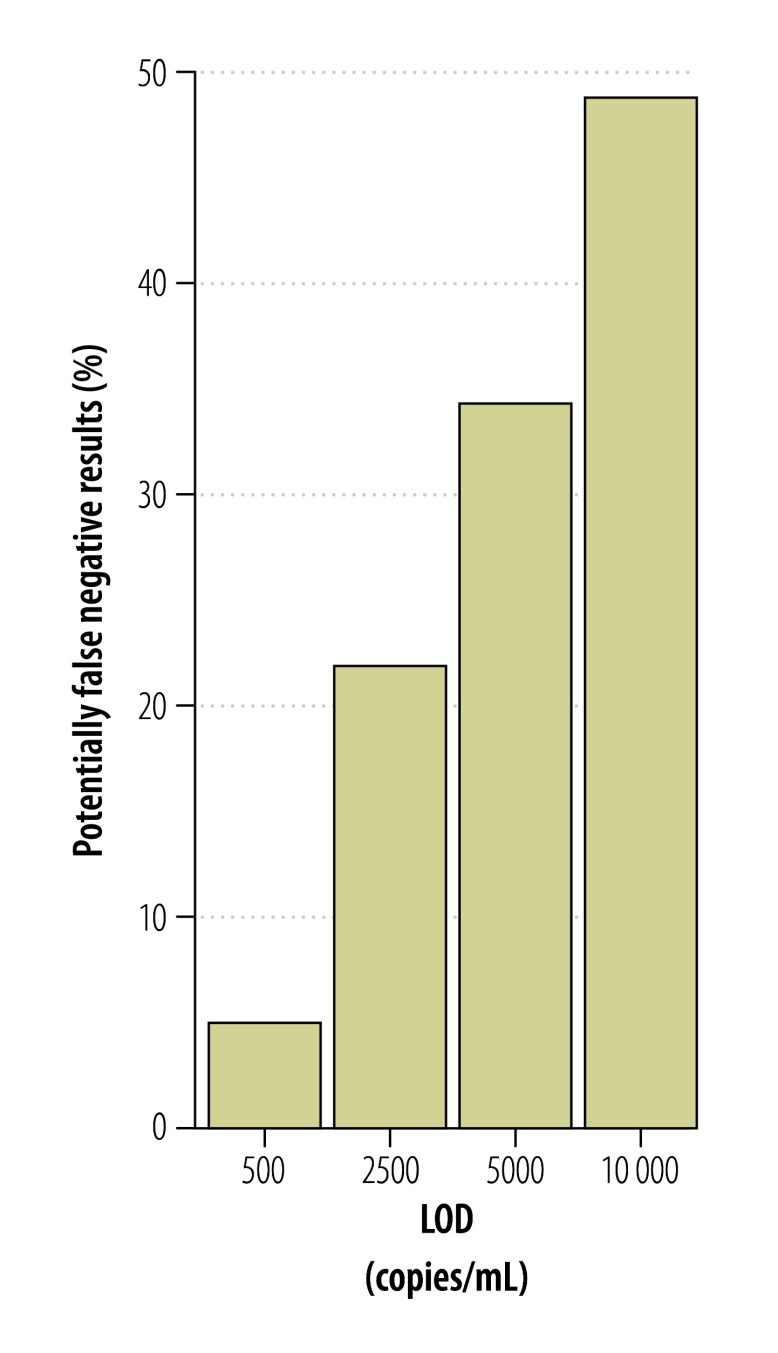
**Risk of false-negative Zika virus test results**

According to preliminary studies, short-term viremia and low viral loads complicate Zika virus detection in blood.^13^^,^^26^ Since Zika virus RNA has been detected in urine from Zika virus-infected patients for two weeks or longer,^26^ we investigated whether blood or urine were the most suitable clinical specimens for diagnoses of acute Zika virus infection. We used the new NS1-based assay to quantify viral loads in 21 serum samples and 12 urine samples from 24 patients infected with virus strains from the current outbreak. As shown in Fig. 7, viral loads generally decreased during 2 to 12 days after symptom onset. Matched urine and blood samples taken on the same day were available from six patients. One patient had a lower viral load in urine, than in blood, two patients had an equal viral load in both samples and three patients had a higher viral load in urine than in blood (Fig. 8). Mean viral loads in all the available clinical samples (including those without known date of symptom onset) were 5x10^4^ RNA copies/mL of blood (range: 1x10^2^–2x10^6^) and 2x10^4^ RNA copies/mL of urine (range: 4x10^2^–8x10^4^). The viral loads in the 24 specimens sampled in the first 12 days after symptom onset were comparable to viral loads in serum from patients sampled during the Zika virus outbreak in the Federated States of Micronesia^13^ in 2007 (Fig. 9). A combined data set of 41 samples comprising the data from the 2007 outbreak (17 samples)^13^ and this study (24 samples) resulted in mean viral loads of 1 × 10^4^ RNA copies/mL of blood (range: 1 × 10^2^–4 × 10^5^) and 5 × 10^3^ RNA copies/mL of urine (range: 4 × 10^2^–6 × 10^4^). The samples were taken during comparable intervals: within 11 days after symptom onset for urine and within 12 days after symptom onset for serum samples. Two of these 41 samples contained viral loads of less than 5 × 10^2^ RNA copies/mL (Fig. 9), leading to an estimated risk of false-negative test results of 5% even when using highly sensitive assays with a technical lower limit of detection of 5 copies/reaction (equivalent to 5 × 10^2^ copies/mL; Fig. 10 and Table 2). Using an insensitive assay with a lower limit of detection of 1 × 10^4^ copies/reaction, the proportion of estimated false-negative test results is about 50% (Fig. 10).

RNA: ribonucleic acid.

Note: Viral loads in different types of clinical specimens plotted per day after symptom onset. The Bonn NS1 assay was used for quantification.

RNA: ribonucleic acid.

Notes: Paired urine and blood samples from three patients taken on the same day within the first 10 days after symptom onset. The Bonn NS1 assay was used for quantification.

Cp/rx: copies/reaction; NS: nonstructural protein; RNA: ribonucleic acid.

Notes: Data from Lanciotti et al. were reported in^13^ and data from the Bonn NS1 assay were generated in this study. Lines in plots show mean viral loads.

LOD: lower limit of detection.

Notes: Projection of false-negative test results according to different lower limits of detection for Zika virus, according to a 2:1 input versus elution volume (e.g. 140 µL blood eluted in 70 µL) as in^13^ and assuming a 100% extraction efficacy.

**Fig. 11:**
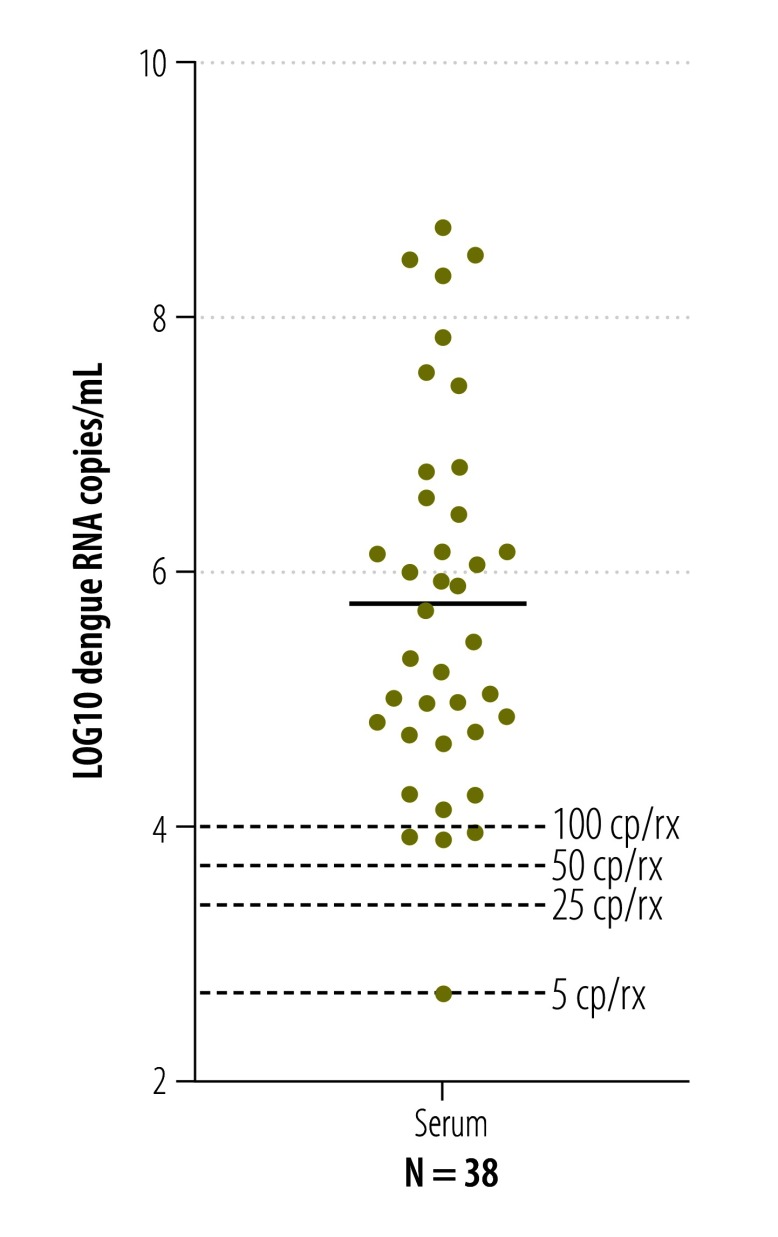
**Viral loads of dengue virus in blood**

**Fig. 12:**
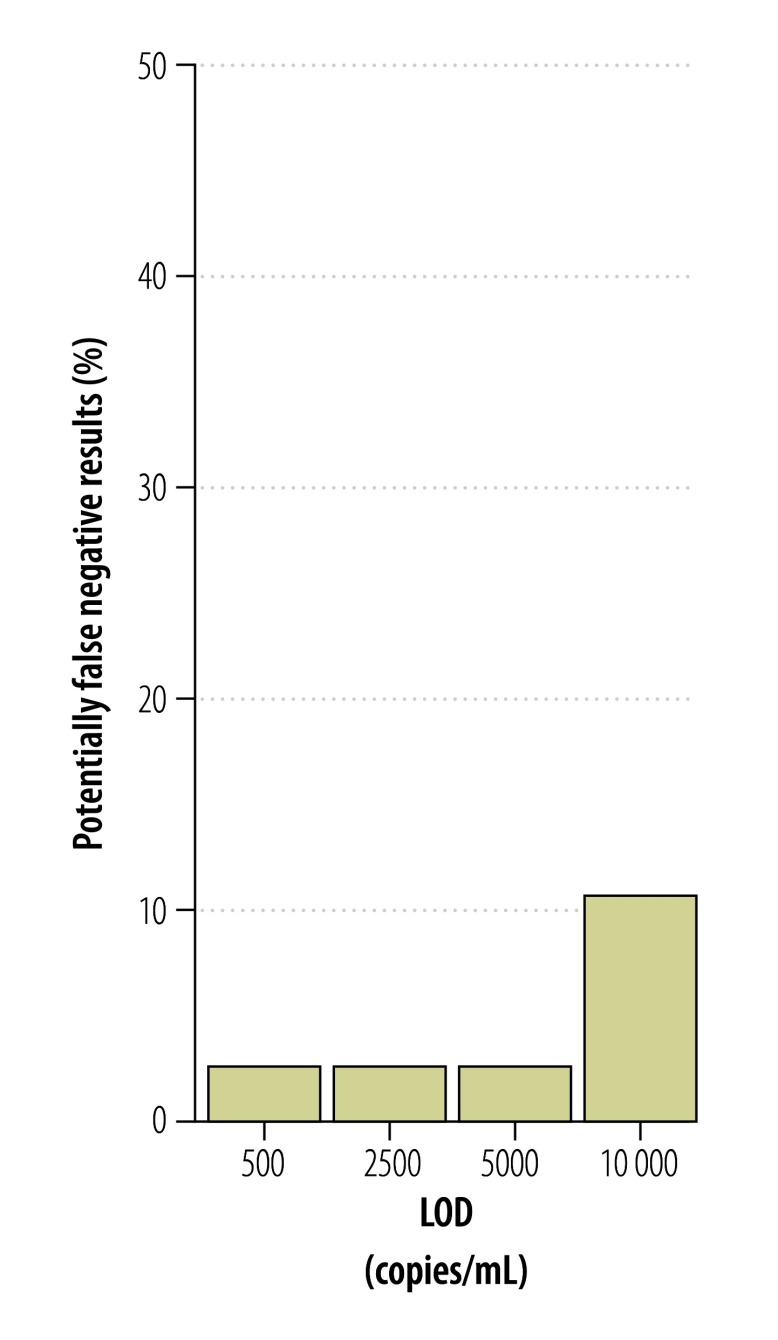
**Risk of false-negative dengue virus test results**

Many of the laboratories in countries affected by the current outbreak that are conducting Zika virus testing also have experience in detecting and quantifying dengue virus. To compare the risk of false-negative test results between these two viruses, we quantified 38 clinical samples positive for dengue virus,^21^ that had been sent to our laboratories for medical diagnostics previously. The mean viral load of dengue was 5 × 10^5^ RNA copies/mL (range: 5 × 10^2^–5 × 10^8^; Fig. 11), meaning that viral loads of dengue in blood were generally about 100-fold higher than viral loads of Zika virus (*t*-test, *P* = 0.03). Accordingly, the estimated risk of false-negative results was several-fold lower for dengue than for the Zika virus (Fig. 10 and Fig. 12).

Cp/rx: copies/reaction; RNA: ribonucleic acid.

Note: Lines in plots show mean viral loads.

LOD: lower limit of detection.

Notes: Projection of false-negative test results according to different lower limits of detection for dengue virus, according to a 2:1 input versus elution volume (e.g. 140 µL blood eluted in 70 µL) as in^13^ and assuming a 100% extraction efficacy.

## Discussion

This study provides guidance on the choice of method for diagnosing Zika virus infections and a molecular control that enables the comparison of results between different laboratories and studies. The low viral loads presented in this and a previous study^13^ suggest a possibility of false-negative test results when using real-time RT–PCR assays in diagnostics.

The results presented here suggest that some published real-time RT–PCR assays may be of limited use for clinical diagnostics during the current Zika virus outbreak. One NS3-based assay that was intended for virus typing should not be used for diagnostics because of its low sensitivity.^7^ Some other assays have features that potentially limit their use in the current outbreak, including limited access to specific probe formats (e.g. the Faye NS5-based assay),^15^ relatively lower analytical sensitivity and high numbers of potential mismatches with members of the Asian Zika virus lineage.^7^^,^^14^ Our novel assays may be more robust against genetic variation, but real-time monitoring of all assays’ oligonucleotide binding regions is required during the current situation. According to our data, the Lanciotti E-,^13^ the Pyke E- and NS1-,^16^ the Bonn E- and the Bonn NS1-based assays are highly sensitive for the Asian Zika virus lineage and show few mismatches within genomic domains targeted by these assays. With the present knowledge of Zika virus variability, we suggest that laboratories can use these five assays for diagnostics during the current outbreak and preferably combine at least two assays to increase clinical sensitivity.^13^

The low viral loads we detected in urine and blood samples are in agreement with two previous studies reporting quantitative data.^13^^,^^26^ In contrast to data from six patients from French Polynesia,^26^ we did not observe a significant difference in the viral loads between the urine and blood samples. Hence, our data do not support urine as a generally more suitable clinical specimen to detect the Zika virus. However, since Zika virus RNA seems to remain detectable in urine and semen longer than in blood,^26^^–^^29^ we suggest that both blood and urine samples should be used for reliable Zika virus diagnostics. The Zika virus has also been detected in saliva,^28^^,^^30^ but we could not evaluate the suitability of saliva samples because we did not have access to such samples.

Although commercial diagnostic real-time RT–PCR reagents for Zika virus detection are available, laboratories in areas affected by the Zika virus outbreak often use non-commercial formulations because of resource constraints.^12^^,^^20^ The non-commercial assays are difficult to standardize and compare. The transfer of essential reagents with coordinated implementation of laboratory protocols and practical training for staff can strengthen accurate real-time RT–PCR diagnostics in resource-constrained settings.^20^^,^^31^^–^^33^ Among the most essential contributions to technology transfer is the provision of standardized control material that can be shipped internationally without biosafety concerns. Research consortia and public health structures can use these reagents to establish a technical basis for test implementation, as demonstrated for severe acute respiratory syndrome and Middle East respiratory syndrome-coronaviruses.^34^^,^^35^ When these viruses emerged, they were novel and new diagnostic tests had to be developed along with the provision of reagents to laboratories. However, for the detection of the Zika virus, where test formulations are already available, assay standardization can only work with the provision of a reference reagent that is universally applicable in all assays, such as our uncRNA reagent or a multicentre validated natural virus standard. Quantitative comparability between studies will enable relative estimates of the transmission risks associated with blood transfusion and solid organ transplantations as well as the transmission risks from body fluids such as semen or saliva. Quantitative data may also shed light on Zika virus pathogenesis, since a higher viral load may be associated with more severe clinical complications, as shown in other arboviral infections, such as those caused by dengue, chikungunya and Crimean-Congo haemorrhagic fever viruses.^36^^–^^38^

Low viral loads in patients imply a high risk of false-negative test results. Until 7 April 2016, only 3% of 199 922 suspected Zika virus cases could be laboratory-confirmed in the PAHO Region.^39^ The low number of confirmed cases could be due to the difficulty in processing high numbers of diagnostic requests in resource-constrained settings. A study from Puerto Rico, showed that 30 (19%) of 155 patients with suspected Zika virus disease could be laboratory-confirmed using molecular and serologic tools.^40^ In addition, a study from Brazil demonstrated that 119 (45%) of 262 patients with suspected Zika virus infection had a positive real-time RT–PCR result.^41^ The higher proportion of laboratory-confirmed cases in those studies and our data suggest that a considerable proportion of patients with low viral loads may have gone undiagnosed by molecular testing during the current outbreak.

Endemic countries also need highly sensitive molecular Zika virus detection methods to ensure safe blood transfusions. The Zika virus has been detected in 3% of blood donors in previous outbreaks^42^ and transfusion-associated transmission has been reported in Brazil.^43^ Our comparison of blood viral loads and real-time RT–PCR sensitivity suggest a risk of false-negative results during pooled and even individual blood donor screening. Such risk has been reported for the West Nile virus, where several people have acquired the virus through blood transfusion or solid organ transplantation, because of false-negative real-time RT–PCR results.^44^

The association of Zika virus infection and fetal malformations demand reliable Zika virus diagnostics for pregnant women.^8^^–^^11^ The current sensitivity of real-time RT–PCR assays suggests that molecular testing during pregnancy may preferentially diagnose highly viremic pregnant women. If intrauterine Zika virus infections and the congenital malformations correlate positively with high Zika virus concentrations, the limited test sensitivity might influence estimates of the manifestation index of congenital disease. The low viral loads in many patients suggest a limited capacity for molecular protocols to exclude Zika virus infection in highly affected areas. Hence, cohort studies investigating Zika virus pathogenesis in pregnant women need to do additional serological testing.

In conclusion, our data emphasize the need for highly sensitive assays in molecular Zika virus diagnostics. In addition to an appropriate choice of method, clinical sensitivity can be increased by testing several specimens per patient, by using more than one real-time RT–PCR target, optimizing RNA purification from clinical samples and by combining molecular and serological testing.^13^ The uncRNA reagent – used as a universal quantitative positive control – can ensure high sensitivity and good comparability of qualitative and quantitative results in diagnostic laboratories and clinical studies.
